# Neuropsychiatric Manifestations of Partial Agenesis of the Corpus Callosum: A Case Report and Literature Review

**DOI:** 10.1155/2019/5925191

**Published:** 2019-03-25

**Authors:** Olusegun Popoola, Olaniyi Olayinka, Heela Azizi, Chiedozie Ojimba, Tasmia Khan, Jisha Kallikkadan, Maleeha Ahmad, Joshua Jay, Cecilia Canale, Sherina Langdon, Alexa Kahn, Deepa Nuthalapati, Sinthuja Jayaraj, Ayesha Mahbub, Olalekan Olaolu, Kodjovi Kodjo, Tolu Olupona, Carolina Nisenoff, Ayodeji Jolayemi

**Affiliations:** ^1^Interfaith Medical Center, New York Department of Psychiatry, Brooklyn, NY, USA; ^2^American University of Antigua College of Medicine, Department of Psychiatry, Interfaith Medical Center, Brooklyn, NY, USA; ^3^Medical University of the Americas, Department of Psychiatry, Interfaith Medical Center, Brooklyn, NY, USA; ^4^Extern, Department of Psychiatry, Interfaith Medical Center, Brooklyn, NY, USA; ^5^St. Matthews School of Medicine, Department of Psychiatry, Interfaith Medical Center, Brooklyn, NY, USA

## Abstract

Agenesis of the corpus callosum is a rare congenital defect that has been linked to psychiatric disorders, cognitive deficits, learning disabilities, and developmental delays. We present the case of a patient with partial agenesis of the corpus callosum who exhibits depressed mood, transient loss of memory, and history of cognitive, social, and behavioral disturbances that developed during his childhood. Recent and pertinent literature was reviewed and the agenesis of the corpus callosum and its associated neuropsychiatric manifestations are discussed.

## 1. Introduction

The implication of the partial agenesis of the corpus callosum in the development and manifestations of a psychiatric disorder is not fully understood. The corpus callosum is the most massive white matter structure in the brain that is involved in the exchange of information between the cerebral hemispheres. The corpus callosum develops between the eighth and 20th week of pregnancy, and its development can be halted during this period [1]. Complete or partial agenesis can occur as part of a syndrome or in isolation [1]. Nonsyndromic isolated agenesis of the corpus callosum can be asymptomatic. However, the prevalence of asymptomatic cases is not known. The incidence of agenesis of the corpus callosum has been reported to be between 0.05 and 0.7 percent of the general population with a male predominance [1]. Despite the proximity of the corpus callosum to the cingulate gyrus, which plays a critical role in the regulation of mood, the implication of its anomaly is yet to be clearly understood. Some neuropsychiatric manifestations such as unipolar depression, bipolar depression, schizophrenia, and severe behavior problems have been linked to the agenesis of the corpus callosum, further making the actual correlation of corpus callosum anomalies to psychiatric disorders unequivocal [2, 3].

These neuropsychiatric disturbances notably observed in patients with corpus callosum anomalies have raised questions regarding the effect of corpus callosum atrophy and agenesis. The current literature demonstrates potential associations between features of psychosis and corpus callosum degeneration or agenesis [4–6]. Previous research has shown some disruption of emotional processing in patients with corpus callosum irregularities [7]. Patients have also been shown to have a potential predisposition to psychosis in the case of agenesis of the corpus callosum, and many have specifically experienced delusions [4, 5]. Though the research in patients with corpus callosum agenesis is expanding, many questions are unanswered. The research highlights some psychotic symptoms and emotional dysregulation but is lacking in outlining common symptomatology of corpus callosum atrophy and agenesis. As of yet, there are no well-defined syndromic classification of corpus callosum pathology. In this report, we aim to discuss corpus callosum pathology as a grouping of specific psychiatric manifestations.

## 2. Case Presentation

We describe the case of a 45-year-old Hispanic man who presented to the psychiatric emergency room on account of depressed mood and forgetfulness. He was found by his niece sitting in the bathroom batting away imaginary flies and crying, stating that he could not remember anything which prompted his niece to call emergency medical services (EMS).

The patient was emotionally labile and could not remember his name or address at the time of presentation. He was hyperverbal and difficult to interrupt, and his speech was disorganized. The patient stated that prior to admission, he left his home and suddenly could not remember how he got to the location he had traveled to. He then returned home and entered the bathroom to look for a belt to hang himself with, because he could not remember any of the evening's events. He stated that he felt lonely and helpless and that he had suicidal thoughts. The patient stated that his sleep had been poor. He endorsed a perceptual disturbance of seeing fleas that were trying to infest his body. He also endorsed an auditory hallucination of a male voice calling his name. Collateral information from his niece, who called the EMS, revealed that the patient had been acting bizarre with two previous episodes of new-onset wandering behavior in the past six months, both associated with heavy alcohol use. She also reported that the patient had a 15-year history of schizophrenia and that he had had similar episodes in the past, which were usually brief and resolved without the need for hospitalization. During a similar episode three years ago, the patient began attacking his family members and was hospitalized after the police were called. The patient also received a diagnosis of major depressive disorder five years ago. The patient was admitted to the inpatient psychiatry unit with a diagnosis of major depressive disorder. Urine toxicology at the time of admission was negative for controlled substances, illicit drugs, and alcohol. The patient's admission Complete Blood Count (CBC) and kidney liver function tests were within normal limits. Rapid regain test was negative. Serum sodium and potassium were 138mmol/L (136–144.0mmol/L) and 4.4mmol/L (3.6–5.1), respectively. Other routine urine analyses and coagulation profiles were also within normal limits as were routine chest radiograph and ECG. Serum thyroid stimulating hormone was below the lower limit of normal 0.409 uIU/ml (0.450–4,500 uIU/ml) and free T4 was 1.09 ng/ml (0.82–1.77 ng/dL). The patient had no symptoms of hyperthyroidism.

**Figure 1:**
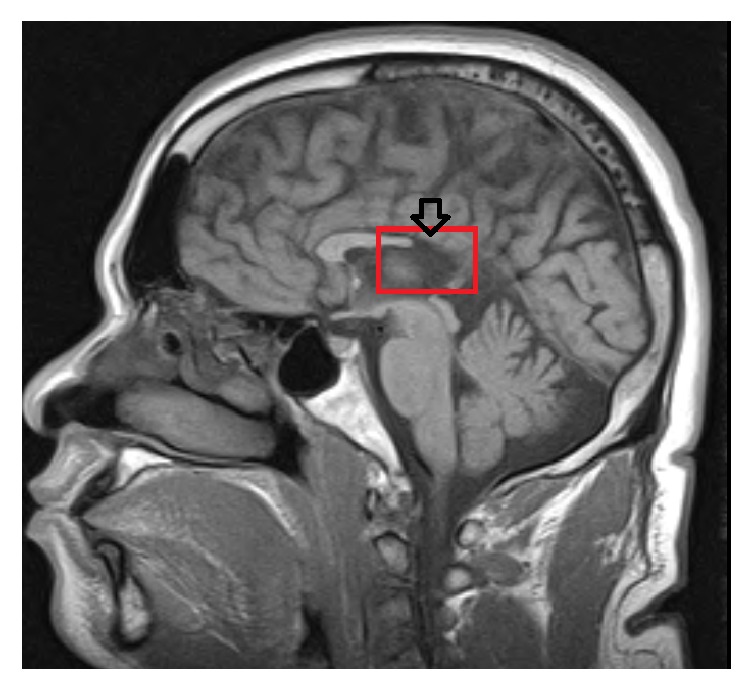
MRI demonstrating partial agenesis of the corpus callosum with the absence of the posterior body and the splenium.

Other chemical laboratory investigations were within normal limits except for dyslipidemia. Computerized tomography (CT) and magnetic resonance imaging (MRI) performed during admission revealed partial agenesis of the corpus callosum with the absence of the posterior body and the splenium as shown in Figure 1.

On day 1 of hospitalization, the patient was hyperactive and restless on the unit. He was treated with escitalopram 10 mg PO daily and risperidone 2 mg PO BID. By day 2 of hospitalization, the patient was able to recall his name and his perceptual disturbances resolved, but he was still hyperverbal, with increased activity. By day 9 of hospitalization, the patient's condition had stabilized, and he was discharged.

According to the patient's mother, the pregnancy was reported to be complicated at five months, and the patient was born at seven months. He had normal gross motor development, but language was delayed until the age of 7 years. The mother reported a history of cognitive developmental delay and intermittent behavioral disturbances that led to his dropping out of school in fifth grade.

## 3. Literature Review

We conducted a literature review to explore various neuropsychiatric manifestations of agenesis of the corpus callosum presented in this case report. This was focused on identifying peer-reviewed articles related to partial agenesis of the corpus callosum and pathology. We searched PubMed, EBSCO, and Web of Science for articles on the function of the corpus callosum and presentation when absent or when there is a lesion without language restriction or restriction to time-period. The search was conducted using the keywords and MeSH terms: “function” “agenesis” “corpus callosum”, “schizophrenia”, and “psychosis.” Screening for eligible articles was conducted independently by six authors. Eligible studies were those that focused on the symptomatology of agenesis of the corpus callosum or defect in the corpus callosum and the respective psychiatric manifestations in human subjects. The citation manager used was Endnote in order to prevent duplication of references.

Because of the rarity of agenesis of the corpus callosum, all types of studies were considered for analysis including experimental, meta-analysis, cohort, case-control, case series, and case reports. The pertinent information from the included articles was abstracted and entered into a data abstraction form using Microsoft Excel constructed by the group. The information extracted from eligible articles includes the title of the article, gender and age of the patient, signs, symptoms elicited, and lab findings/imaging recorded.

A summary of the cases from the review of literature is shown in Table 1. Table 1 covers the already published cases of patients that presented with corpus callosum pathology and their respective symptoms. All the reviewed studies reported the gender of patients with 56 percent being male and 44 percent female. The ages of the subjects ranged from 3 to 73 years; the mean age was 33 years. Furthermore, out of the 15 patient in the reviewed case reports and case series identified to have either a partial or complete agenesis of the corpus callosum about 36 percent were female and 64 percent were male.

Reported symptoms ranged from changes to behavior and personality, including paranoia, hallucinations, and delusions, to cognitive impairment. Radiological findings of the patients varied; however it mainly included agenesis or atrophy of the corpus callosum, followed by lipoma of the corpus callosum.

## 4. Discussion

The case presented is a patient with partial agenesis of corpus callosum with a previous diagnosis of schizophrenia and recurrent transient loss of memory, who presented with mood disorder which resolved within two days during treatment with a combination of antidepressants and antipsychotics. Other probable nonpsychiatric contributors to this presentation were ruled out in the context of normal laboratory findings and the findings from neurologic examination.

Lesions in the corpus callosum have been implied in the etiology of psychiatric disorders; however, the pathophysiologic mechanism is not understood. Studies have shown that the size or thickness of the corpus callosum area is smaller in individuals with schizophrenia when compared to those without schizophrenia [20–22]. Furthermore, a defect in the corpus callosum has been recognized in the neuropathology feature of some neurologic disorders which presents with psychotic symptoms [23, 24].

Research has suggested that ‘‘asymptomatic” individuals with agenesis of corpus callosum (ACC) nevertheless have areas of specific cognitive deficit or learning disability [4]. As of yet, the pattern of consistent cognitive deficits in ACC has not been fully described. It is not clear if the partial ACC in our patient contributed to his cognitive, social, and behavioral disturbances that date back to his childhood. Results from research literature, however, suggest that high-functioning adults with ACC typically have moderate but detectable deficits in the following areas: interhemispheric transfer of complex sensory information and learning; bimanual motor coordination; complex novel problem-solving; processing of subtle phonetic and semantic aspects of language; comprehension of second-order meanings of language; and psychosocial understanding and behavior [4].

## 5. Conclusion

Agenesis of the corpus callosum is a rare condition that is commonly known to be associated with seizures, developmental delays in motor and language skills, and sensory impairment. In addition to these symptoms, there have been multiple findings supporting the association between ACC and neuropsychiatric manifestations such as depression, schizophrenia, bipolar disorder, learning deficits, epilepsy, and conversion symptoms. The findings from our case presentation further support that there is a connection between ACC and the onset of mood and psychotic symptoms. Corpus callosum anomalies interfere with the communication and regulation of activity between the two hemispheres and manifest with these abnormal symptoms. Further studies of mental disorders in patients with ACC are necessary to increase our understanding and awareness of the cerebral basis of many mental health conditions.

## Figures and Tables

**Table 1:** Review of selected literature on cases of patients that presented with corpus callosum pathology.

Article	Gender	Age	Signs and Symptoms	Lab Findings/Imaging Studies
Agenesis of Corpus Callosum and Emotional Information Processing in Schizophrenia [7]	F	23	(i) rituals of washing her hands and clothes out of fear of germs (ii) collected issues from “People” magazine, predominantly of the American singer, Mariah Carey (iii) megalomaniacal delusion in regard to Mariah Carey, with whom she believed was her friend and confidante (iv) persecution belief: everyone around her was conspiring against her, wishing her ill, resulting in her to flee the city to escape her persecutors (v) visual distortions and hallucinations	MRI - partial agenesis of the corpus callosum, above the genu and surrounding the midline, including the isthmus and anterior half of the splenium. Lateral ventricles were large, especially posteriorly, extending into the occipital lobe. fMRI - more lateralized and spatially localized activation, when the emotional content of the stimuli was considered

Delusional Disorder in a Patient with Corpus Callosum Agenesis [8]	F	23	(i) paranoid and persecutory delusions against her brother and mother, speaking ill about her character and conspiring to kill her (ii) delusion of reference (iii) abusive and assaultive, e.g., started breaking expensive household items (iv) poor appetite and difficulty falling asleep, sleeping about 3-4 hours	MRI of brain - agenesis of the corpus callosum, septum pellucidum with bilateral septal colpocephaly

Severe psychiatric disturbance and abnormalities of the corpus callosum: review and case series [5]	Pt 1: F Pt 2: M Pt 3: M Pt 4: M Pt 5: M Pt 6: M Pt 7: F	Pt 1: 35 Pt 2: 29 Pt 3: 41 Pt 4: 33 Pt 5: 22 Pt 6: 12 Pt 7: 31	Pt 1: persecutory delusions, auditory and visual hallucinations, depression, and thought disorder Pt 2: chronic schizophrenia, delusions, thought disorder, auditory and visual hallucinations, and epilepsy Pt 3: manic depressive psychosis and epilepsy Pt 4: acute anxiety state with auditory and visual hallucinations Pt 5: schizoid personality Pt 6: severe behavioral problems Pt 7: personality disorder with depressive and conversion symptoms and epilepsy	Pt 1: CT - lipoma in the posterior corpus callosum. EEG performed twice at age 30; second showed some minor dysfunction Pt 2: CT - agenesis of corpus callosum, left hemi-atrophy of anterior hemisphere due to a CSF-containing frontal lobe cyst, and widened third ventricle. EEG - abnormal with sharp waves over temporal regions Pt 3: CT and MRI - agenesis of the anterior corpus callosum. EEGs - bursts of generalized rhythmic delta activity and left anterior quadrant dysfunction Pt 4: CT - partial agenesis of the anterior corpus callosum. EEGs - normal Pt 5: CT - lipoma in the splenium of the corpus callosum. EEG - slow activity Pt 6: CT - partial agenesis of the corpus callosum with moderately large lipoma, small poorly defined caudate nuclei, and dilated temporal and posterior horns of the third ventricle. EEG - normal Pt 7: CT - agenesis of the corpus callosum and the lateral ventricles were separated by an expanded third ventricle. EEG was normal.

Agenesis of corpus callosum and psychosis - review and case description [9]	N/A	43	Neuropsychological investigation revealed mild mental retardation	CT and MRI - partial agenesis of the corpus callosum EEG - normal

Agenesis of the corpus callosum with associated inter-hemispheric cyst and right frontal pachygyria presenting with psychiatric symptoms in a Kenyan [10]	M	26	(i) depression and recurrence of seizures (ii) normal general and neurological examination apart from some frontal lobe signs on mental status examination (iii) psychosis and labile mood	CT - agenesis of the corpus callosum with inter-hemispheric cyst and right frontal pachygyria. No fat density could be identified in the midline using Hounsfield Units. CBC, EEG - normal

Agenesis of the Corpus Callosum [11]	M	Middle Aged	(i) paranoid delusions: doctors, nursing staff, and patients were against him (ii) auditory hallucinations: voices, both male and female, warning him of the doctors, his wife was being unfaithful, and his death	Imaging - agenesis of the corpus callosum

Acute psychotic symptoms: a manifestation of antiphospholipid syndrome or infarction of corpus callosum [12]	F	25	(i) sleeplessness, excessive talking (ii) persecutory paranoid symptoms (iii) auditory hallucinations, commentary voices and voices conversing with each other	Weighted T2 MRI - sagittal sections showed hyperintense lesions in the splenium of the corpus callosum due to an acute infarction from thrombosis due to antiphospholipid syndrome.

Corpus callosum atrophy and psychosis: a case report [13]	M	31	(i) delusions of persecution	MRI - cerebral atrophy in the frontoparietal zone, non-specific gliotic white matter changes1, and atrophic corpus callosum. Neuropsychological tests (e.g., Stroop test, clock-drawing test) - normal

Cognitive impairments associated with corpus callosum infarction: a ten cases study [14]	10 Pts: M	42-73	(i) impairment of memory, attention, planning, ability to learn new things, orientation, calculation, language, and repetition; No psychiatric manifestations	MRI series (T2W1, T2W2, FLAIR, DWI) - corpus callosum infarction

Cognitive, Behavioral, and Psychiatric Symptoms in Two Children with Agenesis of the Corpus Callosum: Case Report [15]	Pt 1: M Pt 2: F	Pt 1: 11 Pt 2: 10	Pt 1: predominantly visual hallucinations over auditory hallucinations. Appeared tense and confused, with speech impairment. Lacked insight and cognition. Drawings exemplified the presence of anxiety and visual perceptual delays. Pt 2: good personal hygiene, anxious, and mostly coherent speech. No delusions or hallucinations.	Pt 1: normal neurological examination, except for soft neurological signs. EEG - presence of partial complex seizures. Pt 2: Psychological testing - full scale IQ of 102, ATRS score of 28. EEG - intermittent slowing, with occasional high amplitude sharp waves on the left occipital regions. No evidence of clinical seizures. MRI - partial ACC, predominantly the rostrum and the body, and colpocephaly. Thyroid function studies, urine metabolic, amino acid, and organic acid screens were normal.

Developmental Abnormalities of the Corpus Callosum in Schizophrenia [16]	Pt 1: M Pt 2: F	Pt 1: 39 Pt 2: 33	Pt 1: visual and auditory hallucinations, telling him to kill himself, since childhood. Pt 2: Persecutory delusions that led her to attempt to kill herself before her persecutors “dismembered” her and displayed her on the “Johnny Carson Show”. Positive symptoms were refractory to treatment.	Pt 1: T1 weighted MRI - mid-sagittal image, almost complete absence of the corpus callosum Pt 2: T1 weighted MRI - lipoma involving the entire corpus callosum

Agenesis of the corpus callosum and schizophrenia: A case report [17]	M	55	(i) increasingly irritable with persecutory delusions towards his parents (ii) withdrawn, believed to be persecuted as a “homosexual” at work (iii) distressed by persistent auditory hallucinations of several “mumbling” voices which originated from within and outside his body (iv) delusions of reference, believing that people were laughing and staring at him, and television programs referred to him (v) no disturbance of language or mood	CT - absent corpus callosum, midline cystic space extending upward from the third ventricle, and dilated occipital horns of lateral ventricles. MRI - complete absence of the corpus callosum, present anterior commissure, and large CSF filled cavity in the left frontotemporal region, which extends to the vertex, apparently communicating with the ventricular system.

A case of schizophrenia with complete agenesis of the corpus callosum [18]	F	24	(i) auditory hallucinations, voices of angels and the devil (ii) erotic delusions, directed at a male school teacher (iii) other delusions: losing a baby with every menstrual cycle	CT and MRI - complete agenesis of the corpus callosum, with colpocephaly, a sunburst gyral pattern and a high-riding third ventricle.

Corpus Callosum Agenesis and Psychosis in Andermann Syndrome [19]	31 Pts: F 31 Pts: M	3-36	(i) visual hallucinations, persecutory and paranoid type, auditory hallucinations, and paranoid delusions	Unspecified lab/imaging study - 40 of 62 patients presented with corpus callosum agenesis, 35 cases of total and 5 of partial agenesis. 10 of 40 patients presented with posterior fossa atrophy.

Corpus callosum shape alterations in individuals prior to the onset of psychosis [20]	100 Pts: M & F	14-30	(i) 27 out of 100 patients developed a psychotic illness	T1 weighted MRI - Mid-sagittal slice showed reduced thickness of the anterior genu of the corpus callosum in the 27 symptomatic patients

Meta-analysis of magnetic resonance imaging studies of the corpus callosum in schizophrenia [21]	M and F	10-71	(i) signs and symptoms related to disorders in the schizophrenia disorder spectrum or schizotypal personality disorder	MRI - callosal areas of schizophrenic patients over the healthy unrelated control patients, course of the illness influenced the size

The corpus callosum in schizophrenia-volume and connectivity changes affect specific regions [22]	12 pts: M 12 pts: F	29-49	(i) chronic paranoid schizophrenia which was confirmed with structured clinical interviews.	DTI brain image - mid-sagittal slices, corpus callosum segmentation in 3 patients. Volume and fractional anisotropy were positively correlated in both controls and patients. Fractional anisotropy, mean diffusivity, and volume were negatively correlated; however, all three measures were highly correlated with one another. The corpus callosum regions were not all affected equally or the same by the anatomical change.

Marchiafava-Bignami Disease Presenting as Acute Psychosis [23]	M	32	(i) agitation, confusion, delirium, dysarthria, dementia, psychotic symptoms, and other neuropsychiatric manifestations.	CT - hyperdense lesion of the corpus callosum. MRI - hyperintense lesion with restricted diffusion of the corpus callosum.

Clinical and radiological features of Marchiafava-Bignami disease [24]	9 pts: M	37-62	(i) behavioral disorder, seizures, cognitive impairment, delirium, dysarthria and other neurological symptoms.	Pt 1: MRI – extended lesions in the splenium of the corpus callosum. Pt 2: Pt 1: MRI – circumscribed lesions in the splenium of the corpus callosum Pt 3: Pt 1: MRI – extended lesions in the genu, body, and splenium of the corpus callosum. Pt 4: Pt 1: MRI – extended lesions in the splenium of the corpus callosum Pt 5: Pt 1: MRI – ovoid lesions in the splenium of the corpus callosum Pt 6: Pt 1: MRI – extended lesions in the splenium of the corpus callosum Pt 7: Pt 1: MRI - extended lesions in the splenium of the corpus callosum Pt 8: Pt 1: MRI – lobulated lesions in the body, and splenium of the corpus callosum Pt 9: Pt 1: MRI - extended lesions in the splenium of the corpus callosum

“M”, males; “F”, females; “Pt”, patient; “MRI”, magnetic resonance imaging; “fMRI”, Functional magnetic resonance imaging; “CT”, computed tomography; “DTI”, diffusion tensor imaging, “CBC”, complete blood count, “ATRS” abbreviated teacher rating scale, “ACC”, agenesis of the corpus callosum, “EEG”, electroencephalograph, and “IQ', intelligence quotient.
